# Trend of ectopic pregnancy and its main determinants in Hamadan province, Iran (2000-2010)

**DOI:** 10.1186/1756-0500-7-733

**Published:** 2014-10-17

**Authors:** Fatemeh Shobeiri, Najmeh Tehranian, Mansour Nazari

**Affiliations:** Mother and Child Care Research Center, Hamadan University of Medical Sciences, Hamadan, IR Iran; Department of Obstetrics & Gynecoloy, Tarbiat Modarres University (TMU), Tehran, IR Iran; Department of Entomology, School of Medicine, Hamadan University of Medical Sciences, Hamadan, IR Iran

**Keywords:** Ectopic pregnancy, Iran, Women

## Abstract

**Background:**

Ectopic pregnancy remains a major gynaecologyical problem in contemporary gynaecological practice. The purpose of this study was to determine the incidence and some risk factors of ectopic pregnancy in Hamadan province during 2000-2010.

**Methods:**

This was a retrospective descriptive study carried out at hospitals in Hamadan provience, Iran. A total of 872 women with the diagnosis of ectopic pregnancy between 2000 to 2010 were recruited. At initial assessment, 872 medical records were targeted for the assessment, while because of incompleteness of some recorded information, 521 files were finally assessed.

**Results:**

The overall incidence of ectopic pregnancy was estimated to be 2.6 per 1000 recorded pregnancies. While, considerably increased from 1.5 per 1000 pregnancy in 2000 to 4.8 per 1000 pregnancies in 2010. More than half of the women aged 25 to 34 years. 48.2% of selected women were using contraception methods. 5.2%, 14.0% and 5.6% of women had pervious ectopic pregnancy, first or second infertility and pelvic inflammatory diseases, respectively.

**Conclusion:**

Increasing trend of the incidence of ectopic pregnancy is expected due to development and availability of minute diagnostic approaches and also some baseline amendable (contraceptive methods and surgical interventions) and unchangeable (age of pregnancy and residency) parameters.

## Background

Ectopic pregnancy refers to the implantation of a fertilized egg in an abnormal location outside of the uterine cavity, occurring in the fallopian tubes in approximately 97.7% of cases. Other sites includes the cervix, ovary, cornual region of the uterus, and abdominal cavity [1–4]. Since, 1970, the Centers for Disease Control and Prevention (CDC) reported the increasing trend of ectopic pregnancy, however, the case-fatality rate has gradually decreased from 35.5 deaths per 10,000 cases in 1970 to 2.6 per 10,000 cases in 1992 [5–7]. The increased incidence of ectopic pregnancy and in its line, downward trend of its fatality rate has been mainly attributed to improvement of ability in making an earlier diagnosis. Based on the improvement of diagnostic abilities and according to the role of surgical approaches as the main strategy for treatment of this abnormality, physicians would be able to intervene earlier, prevent life-threatening sequelae and extensive tubal damage, as well as preserve future fertility [8]. The first step for optimal management of ectopic pregnancy is identifying major indicators of this abnormality leading increase in susceptibility to this event and its-related complications. Multiple factors contribute to the relative risk of ectopic pregnancy. In theory, all factors that delay the migration of the blastocyst to the endometrial cavity can predispose a woman to ectopic gestation. In this regard, the main risk factors for ectopic pregnancy include tubal damage following infections or any surgical traumas, history of previous ectopic pregnancy, smoking, altered tubal motility, history of infertility, and high maternal age [9–12]. However, various epidemiological assessments result in identifying different natures of the relationship between incidence of ectopic pregnancy and its-related triggering factors. According to this fact that no enough information are available in terms of risk factors for ectopic pregnancy and also according to probable variations in these determinant in different geographical regions, the present study aimed to assess the incidence rate of this gestational abnormality and its main determinants in our province, a great province in western Iran.

## Methods

This was a retrospective descriptive study carried out at hospitals in Hamadan province, Iran. A total of 872 women with the diagnosis of ectopic pregnancy based on quantitative B-hCG and ultrasound) between 2000 to 2010 candidate for medical or surgical treatments were recruited.

During this period, no case of EP was treated in an outpatient setting alone. The information collected for each woman includes sociodemographic characteristics (occupational state, residency: urban or rural area); gynecologic; obstetrics; medical or surgical histories; results of conditions of conception, including contraception and methods used. Clinical signs (abdomen & pelvic pain, amenorrhea, Spotting, vaginal bleeding, gastrointestinal symptoms and dizziness) were extracted from the hospital record files.

At initial assessment, 872 medical records were targeted for the assessment, while because of incompleteness of some recorded information, 521 files were finally assessed. Because of unavailability of information in some record files, the required data were obtained from recorded surgical notes. For determining the incidence of ectopic pregnancy, total number of pregnancies and deliveries were extracted from department of statistics at the deputy of health and medicine of Hamadan. Also, the number of deliveries conducted at home or in labor facilities centers were also collected. According to this fact that the termination of pregnancy includes live birth, fetal death, miscarriage, ectopic and molar pregnancy, the rate of these events were all collected and analyzed. Results were reported as mean ± standard deviation (SD) for the quantitative variables and percentages for the categorical variables. The groups were compared using the Student's t-test or Mann-Whitney U test for the continuous variables and the chi-square test (or Fisher's exact test if required) for the categorical variables. P values less than 0.05 were considered statistically significant. All the statistical analyses were performed using SPSS version 19.0.

## Results

**Figure 1:**
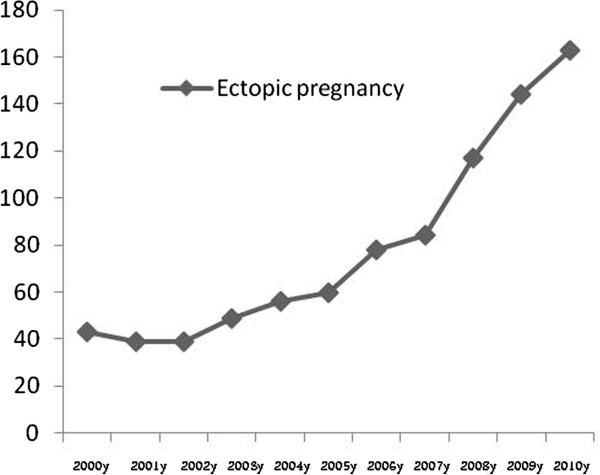
**The trend of the changes in the incidence of ectopic pregnancy (2000-2010).**

**Figure 2:**
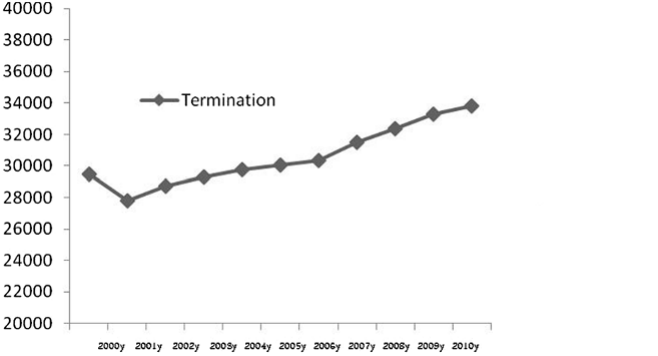
**The trend of the changes in the incidence of termination of pregnancy (2000-2010).**

The details of baseline characteristics are presented in Table 1. During the ten years of assessment (between 2000 and 2010), 323585 pregnancies were recorded in nine great cities of province along with 872 cases of ectopic pregnancies. Thus, the overall prevalence of ectopic pregnancy was estimated to be 2.6 per 1000 recorded pregnancies. The trend of the changes in the number of ectopic pregnancy and pregnancy termination are shown in Figures 1 and 2. The prevalence of ectopic pregnancy was considerable increased from 1.5 per 1000 pregnancy in 2000 to 4.8 per 1000 pregnancies in 2010 with a significant upward trend. With respect to the different determinants of ectopic pregnancy (Table 2), history of previous termination of pregnancy was prevalent in the age range 25 to 34 years (52.2%), over all, 21.8% of the patients had previous history of abortion. Most of them were nulliparous (42.4%), housewives (93.7%), and resided in urban areas (68.7%). Regarding history of pregnancy, 15.9% experienced four pregnancies or more; while 20.6% experienced at least one abortion. Also, 20.0% of women who experienced ectopic pregnancy had irregular menstrual cycles before pregnancy. Regarding used contraception methods, 48.2% of selected women were using contraception methods, whereas the use of IUD, tubulectomy, natural method, and oral contraceptive method were reported in 13.4, 10.4, 9.6, and 7.1%, respectively. Previous history of ectopic pregnancy was revealed in 5.2% of women with the recent experience of this abnormality, and 14.0% expressed to experience infertility. Pelvic inflammatory disorders were reported in 5.6%. Also, 44.5% experienced different types of abdominal or gynecological surgeries out of which 18.9% underwent multiple surgeries.

**Table 1:** **The frequency of total pregnancies, curettage, and ectopic pregnancy in Hamadan province (2000-2010)**

City	Ectopic pregnancy	curettage	Total pregnancy
Hamadan	601	15325	136327
Nahavand	53	3178	32473
Malayer	87	5956	52103
Tuyserkan	31	1591	17877
Kabudrahang	51	1295	15560
Asadabad	24	1286	18633
Famenin	4	8	226
Razan	15	1128	21542
Bahar	6	201	2876
Total	872	29968	327585

**Table 2:** **Baseline information of study population who experienced ectopic pregnancy**

Age group (year)	
15-24	135 (25.9)
25-34	272 (52.2)
35-44	114 (21.9)
Occupational state	
Housewife	488 (93.7)
Employed	33 (6.3)
Residency	
Urban area	358 (68.7)
Rural area	163 (31.3)
Number of Pregnancy	
1	169 (32.4)
2	168 (32.2)
3	54 (10.4)
≥ 4	83 (15.9)
Number of delivery	
0	221 (42.4)
1	163 (31.3)
2	54 (10.4)
≥ 3	83 (15.9)
Number of abortion	
0	408 (78.2)
1	92 (17.7)
2	15 (2.9)
≥ 3	6 (1.2)
Gestational age	
1-2	37 (7.1)
3-4	54 (10.3)
5-6	105 (20.2)
7-8	175 (33.6)
≥ 9	150 (28.8)

Tubal ectopic pregnancy was most common (95.2%) and were frequently right-sided unilateral (52.4%). The most common clinical sign was abdominal and pelvic pain (91.0%) followed by vaginal bleeding (38.6%) and gastrointestinal symptoms (23.6%). Regarding therapeutic management of ectopic pregnancy, 83.9% underwent surgical treatment and 10.7% were medically managed. The most common types of operations were salpingectomy (82.1%) followed by salpingostomy (14.0%). Frequency of main determinants of ectopic pregnancy according age is presented in Table 3. Previous ectopic pregnancy, pelvic inflammation disease, surgery, abortion and contraception methods have shown a significant association with different age. But the clinical signs in different age groups showed no significant difference.

**Table 3:** **Frequency of main determinants of ectopic pregnancy according age**

Determinant factors	Age group (year)			p-value
	15-24	25-34	35-44	
Previous ectopic pregnancy	5 (3.7)	16 (5.9)	6 (5.3)	>0.05*
Previous Pelvic inflammation diseases	12 (8.9)	13 (4.8)	4 (3.5)	>0.05*
Previous surgery	42 (31.1)	124 (45.6)	66 (57.9)	>0.001*
History of abortion	17 (12.6)	65 (23.9)	31 (27.2)	0.009*
**Clinical signs:**				
Abdomen & pelvic pain	125 (26.4)	249 (52.5)	100 (21.1)	0.36
Amenorrhea	10 (23.2)	18 (41.9)	15 (34.9)	0.09
Spotting	35 (32.1)	56 (51.4)	18 (16.5)	0.14
Vaginal bleeding	52 (25.9)	109 (54.2)	40 (19.9)	0.65
Gastrointestinal symptoms	32 (26.0)	55 (44.7)	36 (29.3)	0.05
Dizziness	5 (20.0)	17 (68.0)	3 (12.0)	0.24
**Contraception methods:**				
Hormonal	12 (8.9)	26 (8.9)	12 (10.5)	>0.001*
Tubal ligation	7 (5.2)	19 (14.0)	28 (24.6)	
IUD	19 (14.0)	39 (24.3)	12 (10.5)	
Barrier	97 (71.9)	188 (69.1)	62 (54.4)	

*P>0.05.

## Discussion

Reviewing the trend of the changes in prevalence of ectopic pregnancy showed a rate of 2.6 per 1000 recorded pregnancies with an increasing tend during ten years of study. As previously pointed, the main reason for this upward trend can be due to development of diagnostic tools and also making more direct attention to identifying minute statistics of this phenomenon and its main determinants in health centers in the entire country. According to our observation, the prevalence rate of ectopic pregnancy ranged from 1.5 per 1000 pregnancy in 2000 to 4.8 per 1000 pregnancies in 2010. This might be influenced by some social and epidemiological factors such as age of pregnancy, urban residency, women occupational status, previous surgical operations, and misuse of contraceptive methods. In this regard, the number of parity, previous history of abortion or infertility, and history of pelvic inflammatory disorders were less effective. Reviewing the literatures shows that ectopic pregnancy is a major clinical problem, occurring in 75,000 cases per year in the United States and higher recent observed rate of this phenomenon can be dependant to the development of in vitro fertilization, embryo transfer, microsurgical techniques, and better early diagnosis [13]. The prevalence of ectopic pregnancy among women who go to an emergency department with first trimester bleeding, pain, or both ranges from 6 to 16 percent [14]. The overall incidence of ectopic pregnancy increased during the mid twentieth century, plateauing at approximately almost 20 per 1000 pregnancies in the early 1990s, the last time national data were reported by the Centers for Disease Control [15]. This rising incidence is strongly associated with an increased incidence of pelvic inflammatory disease [16] that was not observed in our study. Another study by Kamwendo and colleagues emphasized the role of pelvic inflammatory disorders on increased prevalence of ectopic pregnancy so that the two to three times higher ectopic pregnancy incidence in women older than 25 years of age was most probably due to a cohort effect as the peak of pelvic inflammatory disease occurred a decade earlier in younger women. Therefore, they concluded that the prevention of pelvic inflammatory disease might not only reduce ectopic pregnancy but also reduce adverse effects on tubal patency [17]. In total, the current incidence of ectopic pregnancy is difficult to estimate from available data (hospitalizations, insurance billing records) because inpatient hospital treatment of ectopic pregnancy has decreased and multiple health care visits for a single ectopic pregnancy have increased [18]. Furthermore, since the incidence is expressed as the number of ectopic pregnancies/1000 pregnancies, the denominator is difficult to determine accurately since early pregnancy failures that do not result in delivery or hospitalization are often not counted [13]. However, some studies believe that current theories concerning the etiology, changes in contraceptive practices, innovations in sterilization procedures, or advances in diagnosis do not appear individually or collectively to explain the increasing incidences reported [19]. In this line, time trends in the age and regional distribution of ectopic pregnancy in some developing countries have suggested that the increasing use of intrauterine contraceptive devices may be a major factor contributing to this recent increase in extrauterine gestations, while, recent age and regional trends in tubal infection appear to be unrelated to the trends in ectopic pregnancy [20, 21]. Hence, controversy has arisen over the best denominator in reporting the incidence of EP. The three commonly used denominators are the number of births, the number of pregnancies and the number of women of reproductive age that has been also previously described [22].

In conclusion, increasing trend of the prevalence of ectopic pregnancy is expected in our region which dependant to both development and availability of minute diagnostic approaches and also some baseline amendable (contraceptive methods and surgical interventions) and unchangeable (age of pregnancy and residency) parameters. The role of pelvic inflammatory disease as a key trigger for ectopic pregnancy was not demonstrated in our survey.

## Conclusion

Increasing trend of the incidence of ectopic pregnancy is expected in our region as a result of the development and availability of minute diagnostic approaches and also some baseline amendable (contraceptive methods and surgical interventions) and unchangeable (age of pregnancy and residency) parameters.
